# Epidermal Growth Factor Receptor Down-Regulation Triggers Human Myoblast Differentiation

**DOI:** 10.1371/journal.pone.0071770

**Published:** 2013-08-15

**Authors:** Marina C. Leroy, Julie Perroud, Basile Darbellay, Laurent Bernheim, Stephane Konig

**Affiliations:** 1 Department of Basic Neurosciences, University Medical Center, Geneva, Switzerland; 2 Department of Clinical Neurosciences, University Hospital, Geneva, Switzerland; University of Minnesota Medical School, United States of America

## Abstract

Initiation of human myoblast differentiation requires a negative shift (hyperpolarization) of the resting potential of myoblasts that depends on the activation of Kir2.1 potassium channels. These channels are regulated by a tyrosine phosphorylation. Using human primary myoblast culture, we investigated a possible role of various receptor tyrosine kinases in the induction of the differentiation process. We found that Epidermal Growth Factor Receptor (EGFR) is a key regulator of myoblast differentiation. EGFR activity is down-regulated during early human myoblast differentiation, and this event is required for normal differentiation to take place. Furthermore, EGFR silencing in proliferation conditions was able to trigger the differentiation program. This occurs through an increase of Kir2.1 channel activity that, via a rise of store-operated Ca^2+^ entry, leads to the expression of myogenic transcription factors and muscle specific proteins (Myogenin, Myocyte Enhancer Factor 2 (MEF2), Myosin Heavy Chain (MyHC)). Finally, blocking myoblast cell cycle in proliferation conditions using a cdk4 inhibitor greatly decreased myoblast proliferation but was not able, on its own, to promote myoblast differentiation. Taken together, these results show that EGFR down-regulation is an early event that is required for the induction of myoblast differentiation.

## Introduction

Skeletal muscle regeneration is a complex process that relies on the presence of satellite cells, the muscle stem cells. Located all along the muscular fibers, between the plasma membrane and the sarcolemma, these myogenic precursors are in a quiescent state. Upon activation, such as with a muscular injury, these cells proliferate as a pool of myoblasts that will first differentiate and then fuse either together to form new fibers or with pre-existing fibers [1]. The myogenic differentiation is a well-coordinated multistep process starting with the drop of the membrane potential of myoblasts. This hyperpolarization induces an increase in the driving force for calcium thus amplifying the influxes requested for the expression of myogenic transcription factors (TFs) [2]–[4]. Two families of TFs are involved in myogenesis: the muscle regulatory factors (MRFs consisting of MyoD, Myf5, Myogenin and MRF4) and the MEF2s [2], [5]–[7].

Myoblast hyperpolarization is the earliest step of the differentiation process known so far. We previously demonstrated that myoblast hyperpolarization relies on an inward rectifier potassium channel, Kir2.1 [8], [9]. Once activated, Kir2.1 channel activity drives the membrane potential from −40 mV to −70 mV. Hinard et al. [10] showed that during myoblast proliferation, Kir2.1 channels are already present at the plasma membrane but not active. Their inactivation is due to a phosphorylation on a specific residue, the tyrosine 242. During differentiation, Kir2.1 channels are rapidly dephosphorylated, leading to their activation. It thus appeared important to evaluate the potential role of receptor tyrosine kinases (RTKs) during the early steps of myoblast differentiation. We focused on the epidermal growth factor receptor (EGFR) as it has been shown to control cell proliferation, survival and motility [11], [12]. Thereby, EGFR has been tremendously studied for its role in cancer development [13], [14]. EGFR overexpression has even been associated with embryonal rhabdomyosarcomas, a type of cancer in which malignant cells arise from muscle precursor cells, and proposed as a biomarker for their diagnosis [15], [16]. Olwin et al. [17] showed that loss of EGFR correlated with differentiation in MM14 cells, although more recent studies in C2C12 cells [18], [19] did not link the EGFR signaling, neither with proliferation nor with differentiation processes. These results suggest that EGFR may regulate myogenesis but its specific role in myoblast differentiation is still poorly understood.

Here, we demonstrate that EGFR activity is sufficient to block differentiation of human myoblasts. Indeed, EGFR silencing was on its own able to induce myoblast differentiation. Key early events of differentiation such as Kir2.1 activation and myogenic transcription factors expression were triggered by EGFR knockdown in proliferation condition.

## Materials and Methods

### Cell Culture

Clonal cultures of human primary myoblasts were prepared from single satellite cells as described previously [20]. Muscle samples were obtained from children during corrective orthopedic surgery. We obtained ethics approval for the study by The University Hospital of Geneva Research Committee on the use of humans as experimental subjects (Protocol 05-078). All human samples were collected anonymously with written consent from the parents of the 5 children involved in the study. Several individual satellite cells (initially randomly chosen) that develop as independent clones of primary myoblasts were used in this study. In all experiments, we used clones between passage 4 and 8 as cells began to become senescent after the 10th passage. All experiments were repeated at least with 3 different clonal cultures in order to ensure the reproducibility of our results. In our paper, “n” numbers refer to the number of clones used. Cells were expanded in growth medium (GM) and differentiated into myotubes in serum-free differentiation medium (DM). GM is a F-10 (Ham)-based medium (Life Technologies) supplemented with Bovine Serum Albumin (0.5 mg/mL, SIGMA-ALDRICH), Fetuin (0.5 mg/mL, SIGMA-ALDRICH), Creatin (1 mM, Fluka), Insulin (0,04 mg/mL, SIGMA-ALDRICH), Dexamethasone (0.39 µg/mL, SIGMA-ALDRICH), Gentamycin (5 µg/mL, Life Technologies), Pyruvate (100 µg/mL, SIGMA-ALDRICH), Uridin (50 µg/mL, SIGMA-ALDRICH), EGF (10 ng/mL, Collaborative Research) and Fetal Bovine Serum (15%, Life Technologies). DM is a DMEM-based medium (Life Technologies) supplemented with Bovine Serum Albumin (0.5 mg/mL, SIGMA-ALDRICH), Creatin (1 mM, Fluka), Insulin (10 µg/mL, SIGMA-ALDRICH), Gentamycin (10 µg/mL, Life Technologies), Pyruvate (100 µg/mL, SIGMA-ALDRICH), Uridin (50 µg/mL, SIGMA-ALDRICH) and EGF (10 ng/mL, Collaborative Research). Specific inhibitors of EGFR tyrosine kinase activity, PD153035 and AG1478 (Calbiochem), were diluted in proliferation medium at 3 µM and 10 µM, respectively. Myoblast protein extracts were obtained using NP40 cell lysis buffer (1% Nonidet NP-40, 50 mM Tris pH 7.4, 250 mM NaCl, 5 mM EDTA) supplemented with phosphatase inhibitors (1 mM Na3VO4, 50 mM NaF and cOmplete Mini protease inhibitor cocktail tablets from Roche).

### Receptor Tyrosine Kinase Assay

The screening of the activated receptor tyrosine kinases was performed with the Human Phospho-RTK array (R&Dsystems) following the manufacturer’s instructions. Briefly, array membranes with the capture antibodies were put in a blocking buffer. Then, they were incubated overnight with the cell lysates (≈ 500 µg proteins). After several washes, arrays were incubated with the detection antibody. Afterwards, membranes were treated with chemiluminescent reagents and exposed to X-ray films for 5 minutes. Pixel density was analyzed with the image analysis software Optiquant®.

### Small Interference RNA (siRNA) Knockdown

Myoblasts were transfected in suspension by incubating the cells in a solution containing 500 µl of Opti-MEM, 3 µl of Lipofectamine RNAiMax (Invitrogen), and 20 pmol of a specific siRNA (Qiagen) according to manufacturer’s protocol (Invitrogen). The optimal number of cells was defined for each experiment so that the confluency was equivalent in any condition. The optimal effect of the knockdown was obtained after 24–48 h post transfection. The siRNA Allstar (Qiagen) was used as a negative control. For EGFR knockdown, the siRNA EGFR10 from Qiagen (TACGAATATTAAACACTTCAA) was used for all the experiments presented here. The siRNA EGFR12 from Qiagen (CAGGAACTGGATATTCTGAAA) was used to confirm the results (data not shown).

### Immunostaining

Cells were fixed with paraformaldehyde 4%, treated with Triton X-100, and incubated in a mix of Tween 20 and goat serum in phosphate-buffered saline (PBS). Mouse monoclonal anti-Myosin heavy chain (1∶500; MF20, Hybridoma bank), mouse polyclonal anti-Myogenin (1∶600; clone F5D, BD Biosciences), rabbit polyclonal anti-MEF2 (1∶600; sc-313, Santa Cruz Biotechnology), and Alexa-Fluor 555 labeled mouse monoclonal anti-Ki-67 (1∶250; BD Pharmingen) antibodies were used. The secondary antibodies used were Alexa-Fluor 488 labeled goat anti-mouse IgG (1∶600; Molecular Probes) and Alexa-Fluor 546 labeled goat anti-rabbit IgG (1∶600; Molecular Probes) together with DAPI (100 ng/ml; Sigma). Scale bars correspond to 20 µm.

### Flow Cytometry

Cells in suspension were kept on ice while fixed with paraformaldehyde 4%. For EGFR labeling, cells were incubated in absence (negative control) or in presence of mouse monoclonal antibody against EGFR (1 µg/100′000 cells; sc-120, clone 528, Santa Cruz Biotechnology) in FACS buffer (PBS 2%FCS). Secondary incubation was performed with Alexa-Fluor 488 labeled goat antibody against mouse IgG (H+L) (1∶400; Molecular Probes). For CD56 labeling, cells were incubated with Alexa-Fluor 488 coupled anti-CD56 antibody (5 µg/100′000 cells, BD Pharmingen) or FITC mouse IgG isotype control (BD Pharmingen). Cell fluorescence (FL1-A) was analyzed with BD Accuri™ C6 flow cytometer. Analysis was done using fluorescence median values. For every condition the control negative value was subtracted and normalization was done with the GM condition.

### Immunoprecipitation

Cells lysates were pre-cleared on protein A Sepharose CL-4B (GE Healthcare) before incubation overnight at 4°C with rabbit anti-GFP (Abcam ab290). Protein A Sepharose beads were added to immunoprecipitate GFP-Kir2.1 fusion proteins (1 hour at 4°C). Washed beads were pelleted by centrifugation (10′000 g for 1 minute), and SDS-β-Mercaptoethanol Laemmli buffer was used to separate immunoprecipitated proteins from the beads (3 minutes at 100°C).

### Western Blotting

Total proteins were prepared in a 5% β-Mercaptoethanol Laemmli buffer, separated on SDS-polyacrylamide gels and transferred to nitrocellulose membranes. Membranes were incubated in TBST (0.1% Tween 20, 20 mM Tris-HCl (pH 7.5), and 137 mM NaCl) and 5% non-fat milk. Blots were incubated with the primary antibodies diluted in 5% non-fat milk or 5% BSA TBST as follows: rabbit monoclonal antibody against EGFR (1∶1500; D38B1 XP®, or 1∶1000; 15F8, Cell Signaling), rabbit polyclonal antibody against MEF2 (1∶3000; sc-313, Santa Cruz Biotechnology), mouse polyclonal antibody against Myogenin (1∶3000; clone F5D, BD Biosciences), mouse monoclonal antibody against α-Tubulin (1∶10000; clone DM1A, Sigma), rabbit polyclonal antibody against Kir2.1 (1∶200; Alomone), mouse monoclonal against phospho-tyrosine (1∶3000; P3300, Sigma) and rabbit monoclonal antibody against phospho-p42/44 MAPK (Erk1/2; Thr2027Tyr204; 1∶1500; D13.14.4E XP®, Cell Signaling). Blots were incubated with horseradish peroxidase conjugated goat anti-mouse or anti-rabbit antibodies (BioRad) diluted 1∶6000. Antibodies were revealed using Western-lightning plus ECL (Perkin Elmer) and Hyperfilm MP (Amersham Biosciences).

### Electrophysiological Recordings

Kir2.1 currents were measured in the whole-cell configuration of the patch-clamp technique at 20–25°C. Signals were sampled at 2.5 kHz, low pass-filtered at 1 kHz and recorded with an Axopatch 200B amplifier (Axon Instruments, Inc.). Pipette resistances were 3–8 megaohms, and compensation was 30–70%. Cell capacitance was obtained from direct reading of the whole-cell capacitance potentiometer of the Axopatch 200B amplifier. To improve the patching procedure, myoblasts were replated 30–60 min before recordings. A current of 0 pA/pF was assigned to myoblasts with a whole-cell current <5 pA. Extracellular solution contained 100 mM *N*-methyl-D-glucamine chloride, 5 mM KCl, 2 mM MgCl_2_, 5 mM Hepes, 50 mM NaOH, 50 mM acetic acid, and 8 mM glucose. The pH was adjusted to 7.4 with Nmethyl-D-glucamine. Intracellular (pipette) solution contained 110 mM KCl, 5 mM NaCl, 2 mM MgCl_2_, 5 mM Hepes, 20 mM BAPTA, 5 mM glucose, and 5 mM MgATP. The pH was adjusted to 7.2 with KOH.

### Cytosolic Calcium Measurements

Myoblasts grown on coverslips were loaded 30 min at room temperature with the cell-permeant fluorescent Ca^2+^ indicator acetoxylmethyl ester Fura-2 (Fura-2-AM) (Biotium Inc., Chemie Brunschwig AG). Fura-2-AM and thapsigargine (Sigma) preparation was done as described previously [4]. Ratiometric images of Ca^2+^ signals were obtained using a Zeiss Axiovert S100 TV microscope (Zeiss AG, Feldbach, Switzerland) equipped with a Cairn Optoscan monochromator (Cairn Research Ltd., Faversham, UK) that rapidly changed the excitation wavelengths between 340 and 380 nm. Fluorescence emissions were captured through a 510WB40 filter (Omega Optical Inc.) using a CoolSnapHQcamera (Photometrics-Ropper Scientific, Tucson, AZ). Image acquisition and analysis were carried out with the Metafluor 6.3r7 software (Molecular Devices, Visitron Systems GmbH, Puchheim, Germany).

### Plasmid Transfection

Myoblasts were transfected using the Nucleofector II (Amaxa, program U-013) with the NHDF Nucleofector Kit (Lonza).

### Statistical Analysis

Results are expressed as mean ± S.E.M of n observations. Student’s *t-*test was used for statistical analyses (asterisk in figures indicates P<0.05). For Western Blots analysis, ImageJ© Software was used to compare band intensities, and Student’s *t*-tests performed (GraphPad Prism© Software asterisks convention: ns p>0.05, * p≤0.05, ** p≤0.01, *** p≤0.001 and **** p≤0.0001).

## Results

### Endogenous EGFR Expression Decreases during Myoblast Differentiation

**Figure 1:**
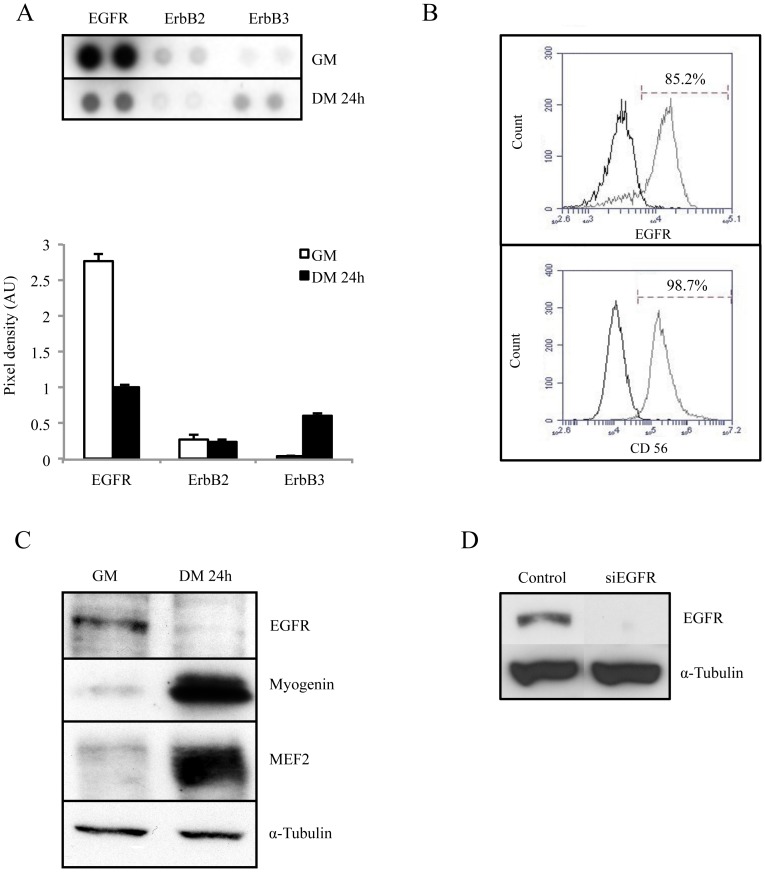
EGFR expression is down-regulated during myoblast differentiation. Cells were cultured in either growth medium (GM) or differentiation medium (DM) for 24 h. **A**. Phospho-RTK arrays were hybridized with 500 µg proteins of whole cell lysates and detected with an anti-phosphotyrosine antibody. Quantification of each spot was performed and expressed in arbitrary units (AU). The results for the ErbB receptors are shown (n = 2). **B**. EGFR and CD56 expression using FACS technique. Myoblasts were fixed and non-permeabilized (n = 5). **C.** EGFR expression was confirmed by Western Blot (whole cell lysates). Myogenin and MEF2 expressions were used as differentiation markers, and α-Tubulin as loading control. **D**. Efficiency of the siRNA against EGFR. Myoblasts were transfected either with a control siRNA or with a siEGFR, and then maintained in proliferation condition for 24 h. EGFR expression was determined by Western Blot (a representative result is shown, n>5).

Kir2.1 channels are regulated by a tyrosine phosphorylation, maintaining them inactive in proliferative myoblasts [2], [10]. We thus looked for a receptor tyrosine kinase (RTK) with a high activity in proliferating myoblasts and whose activity decreases within the first hours of differentiation. For that purpose, a RTK proteome profiler array based on the ELISA technique was used to determine the level of activity of 43 RTKs both in proliferation and differentiation conditions. Among all receptors tested, the epidermal growth factor receptor (EGFR) appeared as the most active in proliferation and, in addition, its activity was clearly reduced after the induction of differentiation. As shown in Fig. 1A, phosphorylation of EGFR was intense in proliferation condition (GM), and decreased by 62% after 24 h of differentiation (DM24h). In comparison, other members of the EGF receptor family, ErbB2 and ErbB3 were poorly active in proliferation (at least 10 times less than EGFR). As expected insulin receptor (IR) and insulin-like growth factor receptor (IGFR) activities were regulated between proliferation and differentiation [21] (Fig. S1). Intriguingly, fibroblast growth factor receptors (FGFRs), which were implicated in the inhibition of rodent myoblast differentiation [17], [22], did not exhibit any detectable activity in human primary myoblasts neither in proliferating nor in differentiating condition. EGFR was detected at the membrane of a large majority of human proliferating myoblasts (83.7±1.6%, n = 5; Fig. 1B). Evaluation of the CD56 marker expression confirmed that 95.7±4.9% (n = 5) of the studied cells were indeed myoblasts. No difference in EGFR expression at the surface of different clones was observed between passages 5 to 7 (n = 11; data not shown). Fig. 1C illustrates that EGFR expression is massively reduced during myoblast differentiation. EGFR was clearly detected in extracts from proliferating myoblasts and below detection in differentiated myoblasts. Expression of Myogenin and MEF2 was used as a control of differentiation. Taken together, these results showed that the endogenous activity and expression of EGFR strongly decreased at the onset of myoblast differentiation, and suggest that this rapid down-regulation of EGFR expression could be involved in the earliest steps of myoblast differentiation. In order to mimic EGFR down-regulation occurring during early differentiation, we used small interfering RNAs (siRNAs) directed against EGFR mRNA (siEGFR). The efficacy of the siRNA to silence EGFR was evaluated by western blot analysis, 48 h post-transfection. Fig. 1D shows that EGFR was below detection in myoblasts transfected with siEGFR. Using this tool, we investigated whether EGFR silencing in myoblasts maintained in proliferation conditions could be associated with a premature differentiation.

### EGFR Silencing Induces Kir2.1 Channel Activation, a Molecular Event Linked to Early Differentiation

**Figure 2:**
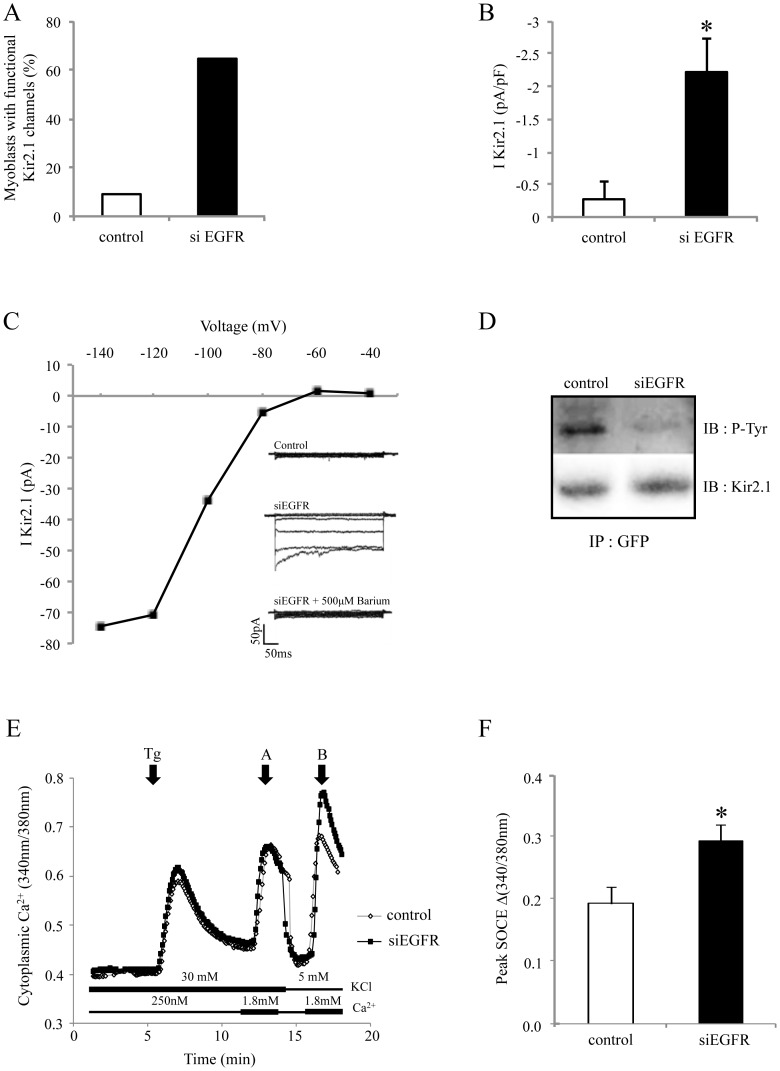
Kir2.1 is activated by EGFR knockdown in myoblasts. Myoblasts were transfected with either control siRNA or siEGFR. **A**. Percentages of transfected myoblasts with functional Kir2.1 channels, 48 hours post-transfection. **B.** Current densities of the total population of myoblasts (including myoblasts with no current, i.e. <5 pA). **C.** Current-voltage relationships of a myoblast transfected with siEGFR. Voltage-steps were to −40, −60, −80, −100, −120 and −140 mV from a holding potential at −60 mV. The inset shows a control myoblast with no Kir2.1 current, a typical Kir2.1 current recorded from a myoblast, 48 h after transfection with siEGFR. Addition of 500 µM Ba^2+^ inhibited this current. **D.** Myoblasts were first transfected with control siRNA or siEGFR, and 24 h later with a plasmid coding for GFP-Kir2.1. One day after, immunoprecipitation of GFP was performed. Immunoblots reveal Kir2.1 and phospho-tyrosine (P-Tyr). **E**. Cytoplasmic Ca^2+^ was assessed with Fura-2-AM on proliferating myoblasts, 2 days after siRNA transfection. Intracellular Ca^2+^ stores were depleted with 10 µM thapsigargin (Tg) in a medium containing 250 nM Ca^2+^. Then 1.8 mM Ca^2+^ was subsequently added to reveal SOCE. The first part of the experiment was performed with a medium containing 30 mM KCl in order to clamp cells at around −40 mV. The second part was performed with a medium containing 5 mM KCl allowing cells to hyperpolarize. **F**. Quantification of peak SOCE (n = 6; * p<0.05).

We previously defined Kir2.1 channel activation as one of the earliest events of myoblast differentiation [2]. As previously demonstrated [2], [10], in control condition, only 9% of proliferating myoblasts exhibited Kir2.1 activity and, in these cells, Kir2.1 current density was low (Kir2.1 measured at −120 mV: −0.3±0.27 pA/pF, n = 11; Fig. 2AB). Interestingly, while still in proliferation condition, 64% of siEGFR-transfected myoblasts exhibited typical large Kir2.1 currents with current densities comparable to those obtained with myoblasts induced to differentiate for 24 hours (measured at −120 mV: −2.2 pA/pF±0.5; n = 18; Fig. 2AB) [2], [8], [10]. As expected, addition of 500 µM barium completely blocked this current, and the current-to-voltage relationships exhibited the classic inward rectification of Kir currents (Fig. 2C). In agreement with our previous results [10], activation of Kir2.1 channels should result from a decrease of the Kir2.1 channel tyrosine phosphorylation. Fig. 2D shows that, indeed, silencing EGFR reduces Kir2.1 tyrosine phosphorylation.

Kir2.1 channel activation is responsible for a myoblast hyperpolarization induced in the early steps of differentiation [9]. This hyperpolarization from around −40 mV to −70 mV generates a calcium entry through store-operated calcium channels and, thereby, favors differentiation [3], [4]. Accordingly, in EGFR-silenced proliferating myoblasts (expressing Kir2.1 channel and thus hyperpolarized at −70 mV), a larger store-operated calcium-entry (SOCE) should be observed. In Fig. 2EF, SOCE was induced by a thapsigargin application to deplete calcium stores, and assessed (using Fura-2-AM) during re-addition of extracellular calcium (1.8 mM). In our protocol, calcium re-addition was performed twice. The first calcium re-addition (arrow A) was made in presence of 30 mM external potassium. This high potassium concentration was chosen as it precludes the hyperpolarization to −70 mV and, according to the Nernst equation, maintains the resting potential around −40 mV. It can be seem that, in myoblasts not able to hyperpolarize, SOCE was not affected by EGFR silencing. On the other hand, when calcium was re-added (arrow B) in presence of a potassium concentration (5 mM) allowing the hyperpolarization to −70 mV, SOCE was clearly larger in EGFR-silenced myoblasts. This result suggests that silencing EGFR triggers a hyperpolarization in myoblasts that can increase calcium entry.

### EGFR Silencing Triggers Myoblast Differentiation and Fusion

**Figure 3:**
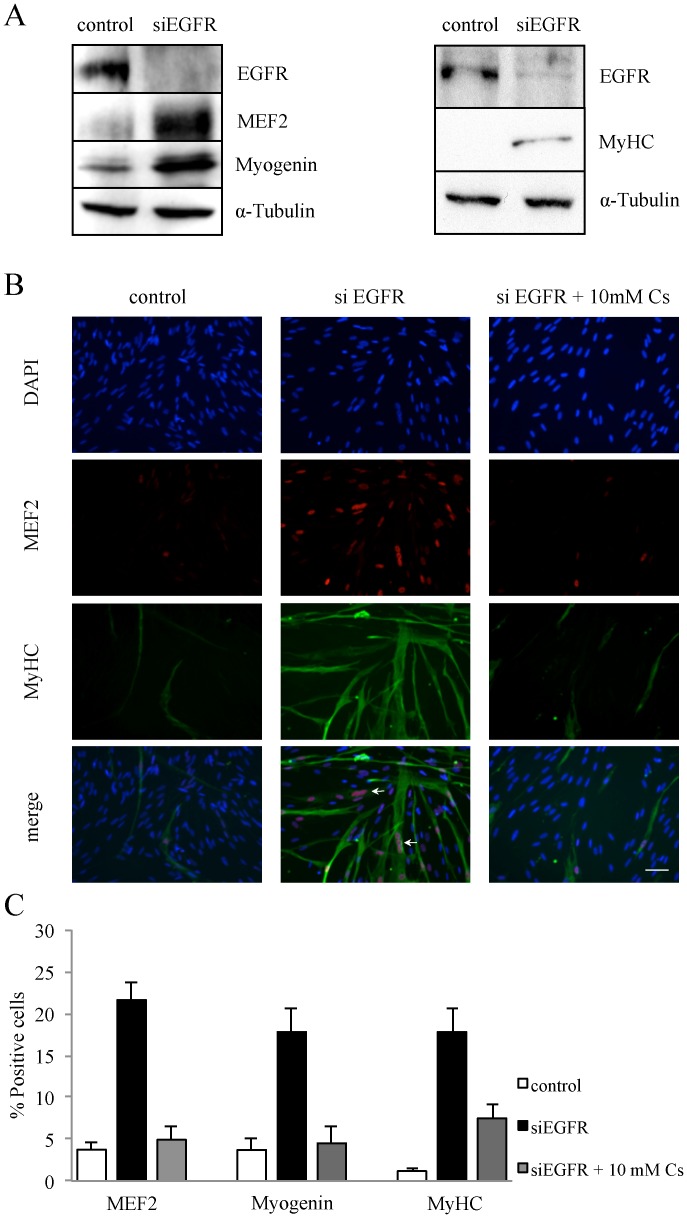
Myoblast differentiation is induced by EGFR knockdown. Proliferating myoblasts were transfected either with a control siRNA or with a siEGFR, and then maintained in proliferation condition for 5 days. **A.** Total cell lysates were analyzed by Western Blot. Myogenin and MEF2 expressions were used as early markers of differentiation, Myosin heavy chain (MyHC) as a late marker of differentiation, and α-Tubulin as a loading control. **B**. Differentiation of myoblasts was observed by the staining of MEF2 (red) and MyHC (green), and nuclei by DAPI (blue). Cesium (Cs 10 mM) was added to the medium to block the hyperpolarization. Myotubes resulting from myoblast fusion were observed (white arrows). **C.** Quantification of the immuno-fluorescence shown in B (n>3).

As the Kir2.1-induced hyperpolarization is essential for the initiation of myoblast differentiation [2], we investigated in proliferating myoblasts the effect of EGFR silencing on the expression of Myogenin and MEF2, two well-known early myogenic markers, and MyHC (Myosin heavy chain), a late muscle-specific protein. As shown in Fig. 3A, proliferating myoblasts silenced for EGFR clearly expressed these three markers of differentiation. Expression of these proteins after EGFR knockdown was confirmed by immuno-fluorescence (Fig. 3B and quantification in Fig. 3C). MEF2 expression increased 5.7 fold, Myogenin 4.8 fold and MyHC 15 fold after EGFR silencing in myoblasts. It is important to note that in myoblasts transfected with siEGFR, the MyHC staining revealed multinucleated myotubes demonstrating that EGFR knockdown induced myoblast differentiation in growth medium (Fig. 3B, white arrows). Addition of 10 mM cesium, a known inhibitor of potassium channels that we previously used to inhibit myoblast hyperpolarization to −70 mV [2], counteracted the effect of EGFR knockdown on differentiation. These results were obtained 5 days post siEGFR transfection, while Fig. 2 shows that Kir2.1 channels are activated within 24 to 48 hours post transfection. We thus assessed the expression of the early marker MEF2 48 hours after siEGFR transfection. We found that, 48 hours post transfection, only 8% of EGFR-silenced myoblasts expressed MEF2 (not shown), whereas 60% of EGFR-silenced myoblasts expressed Kir2.1 channels (Fig. 2). Hence, activation of Kir2.1 channels does not result from, but precedes the induction of the myogenic program of differentiation.

### EGFR Activity Decreased during the Early Steps of Myoblast Differentiation

**Figure 4:**
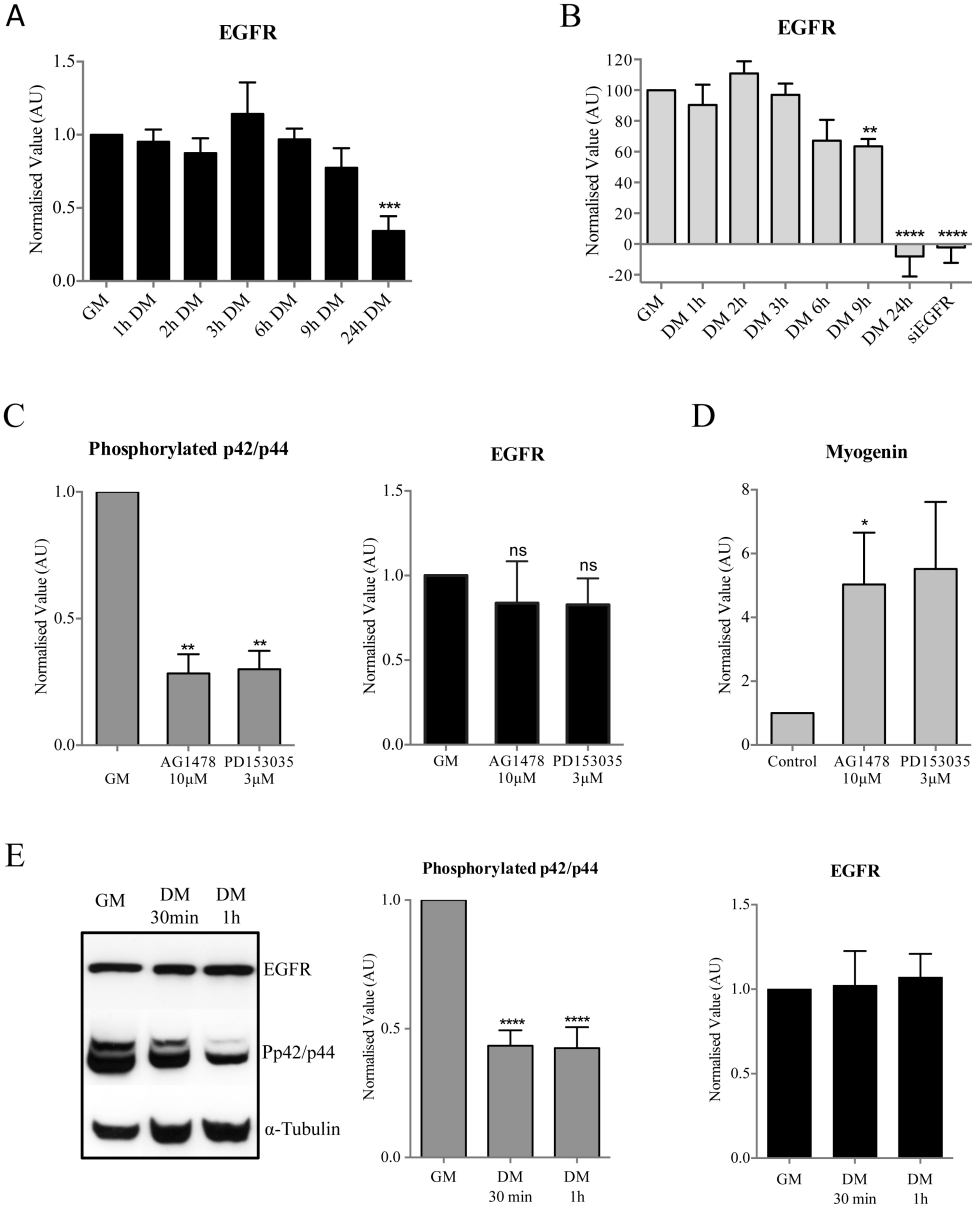
Modulation of EGFR activity during the first hours of myoblast differentiation. All results were normalized to the result obtained for GM conditions. **A**. Myoblasts were lysed in proliferation (GM) and at different times in differentiation conditions (DM). Western blot analysis of EGFR on whole cells extracts. EGFR expression decreases by 64% after 24 h in DM (n = 5). **B**. Myoblasts were fixed in proliferation (GM) and at different times in differentiation conditions (DM). Flow cytometry was performed on non-permeabilized myoblasts. For the control condition myoblasts were only incubated with the secondary antibody. A significant decrease of EGFR at the plasma membrane is observed after 9 h in DM (n = 5). To control the specificity of the antibody against EGFR, we verified that the antibody did not bind any more to myoblasts transfected with a siEGFR. **C-D-E**. Total EGFR, phospho-p42/p44 MAPK and Myogenin expressions were assessed by Western blot. α-Tubulin expression was used as a loading control. **C.** Efficiency of EGFR inhibitors. Myoblasts were cultured in GM for 1 h with AG1478 at 10 µM or PD153035 at 3 µM. Western blot analysis shows a significant decrease of phospho-p42/p44 MAPK expression but no difference of EGFR expression (all n = 3). **D**. Myogenin expression after 24 h treatment with EGFR inhibitors in proliferating conditions (n = 4). **E**. Phospho-p42/p44 MAPK and EGFR expression during the first hour of differentiation (n = 5).

According to our results, EGFR expression or activity should decrease before Kir2.1 activation occurs. We already published that Kir2.1 activation occurs approximately after 6 hours in differentiation conditions [10]. Western blot analysis showed that total EGFR expression was stable during the first 9 hours of differentiation, and a decrease of EGFR expression was only observed after 24 hours (Fig. 4A). We also investigated EGFR localization at the plasma membrane during differentiation using Flow Cytometry as receptor inactivation is known to be regulated by internalization. Membrane expression of EGFR was stable during the first 6 hours of differentiation and only began to decrease significantly after 9 hours (p = 0.0011, Fig. 4B). After 24 hours in differentiation conditions, EGFR was not present at the membrane anymore.

EGFR activity is associated with the activation of diverse downstream pathways, among them the p42/p44 MAPK (extracellular signal regulated kinase (ERK1/2)) pathway [23]. We used two inhibitors of EGFR activity (AG1478 and PD153035) to determine the importance of EGFR activation on the p42/p44 MAPK pathway in human primary myoblasts. Fig. 4C (left) shows that both inhibitors strongly decreased the p42/p44 MAPK activity (assessed by the phosphorylation state of p42/p44 MAPK) after one-hour treatment (72% and 70% decrease for AG1478 and PD153035 respectively). This inhibition was maintained after 24 hours (78% and 69%, data not shown). We verified that EGFR stability was not affected by the inhibitors (Fig. 4C, right). Together, these results strongly suggest that the activation of p42/p44 MAPK pathway in human primary myoblast is mainly controlled by EGFR. Consistently, pharmacological inhibition of EGFR activity for 24 hours induced Myogenin expression in proliferation conditions, which confirms our results obtained using siEGFR.

We then assessed the phosphorylation state of p42/p44 MAPK to evaluate EGFR activity during the very early step of myoblast differentiation. Fig. 4E shows that, although EGFR expression was stable, p42/p44 MAPK activity decreased by 57% during the first hour of differentiation. Taken together, our results show that EGFR activity decreases rapidly after induction of differentiation and precedes Kir2.1 channel activation that takes place after 6 hours in differentiation conditions [10].

### EGFR Activity Inhibits Myoblast Differentiation

**Figure 5:**
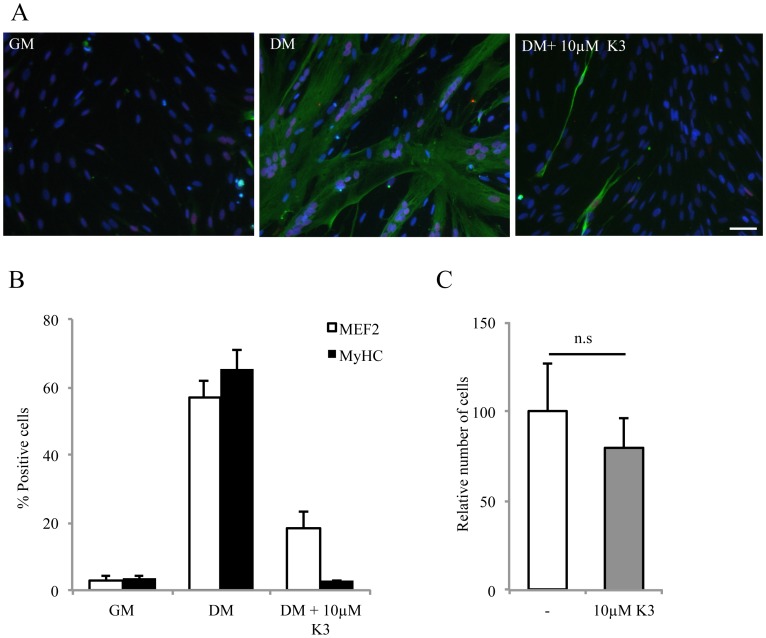
Vitamin K3 prevents differentiation without inducing proliferation. A. Myoblasts were cultured either in GM or in DM ±10 µM vitamin K3. Differentiation was assessed by immunostaining (MEF2 in red; MyHC in green; nuclei in blue with DAPI). B. Quantification of the immuno-fluorescence shown in A (n = 4). C. Vitamin K3 in differentiation conditions for 24 h does not increase cell proliferation as assessed by the average of the number of nuclei (DAPI) per field (from 3 independent experiments; p = 0.4).

Our results so far suggest that one of the physiological roles of EGFR in proliferating myoblasts is to inhibit differentiation. Accordingly, induction of myoblast differentiation should be prevented by EGFR activity. To test this point, we transfected human myoblasts with a plasmid encoding either the fusion protein EGFR-EGFP [24] or the empty vector encoding EGFP. However, while 87%±4 of myoblasts were positive for EGFP after transfection of the empty vector (data not shown, n = 3), only 8%±4 of the myoblasts were positive for EGFP after transfection of the plasmid encoding the fusion protein EGFR-EGFP. In addition, the level of EGFR expression, assumed by the fluorescent level of the EGFP, was very low in these positive cells. These results suggest that EGFR expression in human primary myoblasts is highly regulated at the protein level, precluding unfortunately any EGFR over-expression experiment. To circumvent this problem we used vitamin K3. Abdelmohsen et al. [25] showed that vitamin K3 (Menadione) could, by blocking the activity of phosphatases that dephosphorylate EGFR, maintain the receptor in an active state. Fig. 5A and B shows that, after 48 hours in differentiation conditions, control myoblasts clearly expressed the early and late markers of differentiation, MEF2 and MyHC. As predicted, addition of 10 µM vitamin K3 strongly prevented MEF2 and MyHC expression. In control cultures, 57%±4 and 65%±5 of myoblasts expressed MEF2 and MyHC whereas, in vitamin K3-treated cultures, only 18%±5 and 3%±0.4 of myoblasts expressed MEF2 and MyHC (p<0.01; n = 4). As shown in Fig. 5C, the number of cells in vitamin K3-treated conditions is not significantly different from that of control conditions. This observation shows that EGFR activity inhibits myoblast differentiation by mechanisms independent from the activation of proliferation.

### Cell Cycle Arrest does not Trigger Myoblast Differentiation

**Figure 6:**
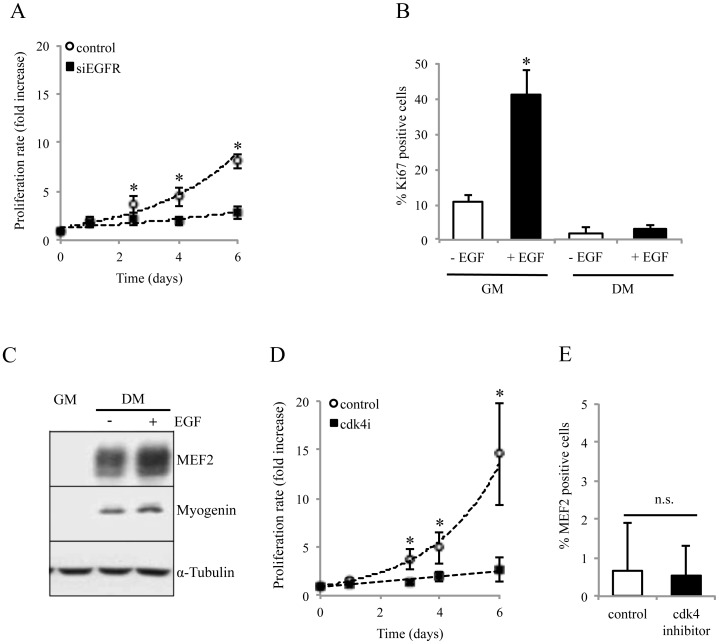
EGFR signaling stimulates myoblast proliferation. **A/D.** Myoblasts proliferation was inhibited after transfection with siEGFR and by 600 nM of the cdk4 inhibitor (Calbiochem). Proliferation was assessed using Ki67 immunostaining. (n = 3; * p<0.05). **B**. EGF stimulates myoblast proliferation in growth but not in differentiation medium. Myoblasts were cultured 24 h in GM or DM complemented or not with 10 ng/mL of EGF. **C.** EGF does not block myoblast differentiation in differentiation medium. Myogenin and MEF2 expressions were used as differentiation markers, and α-tubulin as loading control. **E.** Addition of 600 nM of cdk4 inhibitor in proliferation condition does not induce myoblast differentiation, assessed by MEF2 expression (n = 3).

EGFR is a growth factor receptor known to be implicated in the proliferation of many cell types [26], [27]. Indeed, six days after transfection, EGFR-silenced myoblasts only multiplied by 2.7, whereas control myoblasts multiplied by 8 (Fig. 6A). We also confirmed, using Ki67 expression, the mitogen effect of EGFR activity on proliferating myoblasts by increasing EGF in the growth medium. Our growth medium contains 15% fetal calf serum and thus an unknown amount of EGF [20]. As expected, addition of 10 ng/ml EGF in the growth medium increased the percentage of Ki67 positive myoblasts from 10% to 40% (Fig. 6B). In contrast, the same concentration of EGF had no effect on the proliferation rate of myoblasts when added in the differentiation medium (DM with EGF: Ki67 = 2.95%, DM without EGF: Ki67 = 1.95%). Furthermore, as shown in Fig. 6C, the presence of EGF in differentiation medium does not inhibit myoblast differentiation. These results confirm that the mitogenic activity of EGF is restricted to proliferating myoblasts.

Finally, we excluded that the effect of EGFR silencing on differentiation could be a consequence of the cell cycle arrest. It is known that myoblasts induced to differentiate stop their cell cycle in G1 [28] [29], [30]. We thus used the cdk4 cell cycle inhibitor as it specifically stops cells in G1 phase. As expected this inhibitor strongly affected proliferation (Fig. 6D and data not shown for other concentrations of cdk4 inhibitor). However, blocking the cell cycle did not induce any differentiation, as the percentage of MEF2 positive cells was unaffected by 5 days of cdk4 inhibitor treatment (Fig. 6E). This result shows that myoblast cell-cycle arrest does not trigger myoblast differentiation. We concluded that the induction of myoblast differentiation, observed in EGFR down-regulated myoblasts, does not result from the inhibition of myoblast proliferation.

## Discussion

Our present work shows that EGFR plays a major role during the onset of human myoblast differentiation. EGFR activity is essential for the inhibition of the differentiation process in proliferating myoblasts. EGFR silencing in myoblasts kept in proliferation condition not only drastically reduces their proliferation but also induces their differentiation. We could, in addition, show that EGFR activity is physiologically downregulated during the differentiation of human primary myoblasts, and that this down-regulation is an important trigger of the differentiation process.

We have previously identified the activation of Kir2.1 channels as an essential step for myoblast differentiation and fusion, and proposed that the Kir2.1-induced hyperpolarization is one of the earliest triggers known so far [2]. This model is supported by the fact that the activation of Kir2.1 currents during myoblast differentiation precedes and is required for the expression of early markers of differentiation, the transcription factors Myogenin and MEF2. We demonstrate here for the first time that EGFR silencing triggers Kir2.1 channels dephosphorylation, activation, and, in agreement with our previous results, that this activation is followed by myoblast differentiation. We also show that myoblast differentiation generated by EGFR silencing is inhibited by cesium ions, a Kir2.1 channel blocker. Finally, we provide evidences that SOCE is enhanced after EGFR invalidation, and that this effect is abolished when myoblasts are maintained in a depolarized condition. Altogether, this strongly suggests that the induction of myoblast differentiation by EGFR down-regulation relies on the Kir2.1-linked hyperpolarization together with the induced cell-cycle arrest.

EGFR inhibition led to the down-regulation of the p42/p44 MAPK pathway. Thus, in addition to its effect on Kir2.1 channels, EGFR is also involved in the human myogenic program by down-regulating the p42/p44 MAPK pathway. In myoblasts, this pathway has been shown to be involved in proliferation, migration, and repression of the myogenic program [31]–[33]. In agreement with us, Wu et al. observed that, at the onset of differentiation, a decrease of p42/p44 MAPK activity upon serum removal is needed for differentiation to take place [34]. However, its re-activation seems required for efficient myoblast late differentiation and fusion [35], [36]. Consistently, we observed that p42/p44 MAPK activity reappeared during later stages of human myoblast differentiation (data not shown). Taken together, these results highlight a multiple role for the p42/p44 MAPK pathway in the myogenic differentiation program.

The mitogenic effect driven by EGFR signaling is well established for many cell types. Golding et al. [37] showed that members of the EGFR tyrosine kinase family (EGFR, ErbB2 and ErbB3) appear on myofiber-associated satellite cells after their activation during skeletal muscle regeneration. Thus a necessary control was to establish that inhibition of human myoblast proliferation by itself could not induce myoblast differentiation. Our experiments with cell cycle inhibitor confirmed that inhibition of proliferation, while necessary, is not sufficient for the induction of myoblast differentiation. From these results, we conclude that EGFR down-regulation has two distinct roles: (i) cell cycle entry/arrest and (ii) induction of myoblast differentiation via at least the activation of Kir2.1 currents and the down-regulation of p42/p44 MAPK pathway.

In proliferation, when EGFR is highly active, Kir2.1 channels are maintained inactive by the phosphorylation of Tyr242 [10]. Activation of Kir2.1 currents takes place after 6 hours in differentiation medium [2], when EGFR is no longer active. The inhibition of Kir2.1 currents by EGFR could be direct or indirect. Wischmeyer et al. [38] showed that the inhibition of Kir2.1 via the phosphorylation of Tyr242 in a transformed human kidney cell line is modulated by growth factors such as EGF. In addition, Sun et al. [39] demonstrated, in human bladder urothelial cells, that addition of EGFR ligands (EGF and HB-EGF) induced the phosphorylation of Kir2.1 channels, resulting in a decrease of their conductance. On the other hand, while others and we described that Kir2.1 currents are inhibited by a tyrosine phosphorylation, other studies suggest that Kir2.1 currents can be activated by a tyrosine phosphorylation. Most of these works are based on the effect of genistein, a commonly used tyrosine kinase inhibitor. Genistein inhibited Kir2.1 currents in rat ventricular myocytes [40] and in rat osteoclasts [41]. Chiang and al. proposed however that genistein directly blocks Kir2.1 currents (without involving a tyrosine kinase) in Guinea pig ventricular myocytes [42]. Additionally, it has been demonstrated that genistein can act directly on the transmembrane and the pore region of Kir2.3 [43]. Nevertheless, more recently, a potent inhibitor of EGFR, AG556, has been shown to block Kir2.1 activity in HEK293 cells via a tyrosine kinase inhibition [44]. The EGFR kinase activity was proposed to activate Kir2.1 currents through the phosphorylation of Tyr242, a result that is on contradiction with ours. Thus, it seems that Tyr242, once phosphorylated, may interact with different proteins able to either activate or inactivate Kir2.1 currents. Hence, while Kir2.1 regulation by tyrosine kinases and particularly EGFR seems obvious, whether it activates or inhibits the channels is still unclear and likely depends on the cellular model. In human myoblasts, we showed that EGFR promotes Kir2.1 inactivation, and is a keystone of the differentiation process.

In this study, we demonstrated that EGFR activity and expression is endogenously regulated in human myoblasts. Our current understanding is that this down-regulation triggers myoblast differentiation via the activation of Kir2.1 channels. As described in different models, degradation of receptors tyrosine kinase, and especially EGFR, is one of the mechanisms used by the cells to negatively regulate the proliferation induced by growth factors [45]. However, what controls EGFR activation and inactivation/degradation in proliferating or differentiating myoblasts remains to be elucidated.

## Supporting Information

Figure S1 **Receptor Tyrosine Kinases (RTKs) were tested for their activities in proliferation (GM) and differentiation conditions (DM 24 h).** Panel A: Immunoblots of the RTK profiler array. Each pair of spots corresponds to a phosphorylated RTK. Panel B: List of the RTKs tested.
(TIF)
