# Supplementary figures and images for: Epidermal Growth Factor Receptor Down-Regulation Triggers Human Myoblast Differentiation
Source: PLoS One. 2013 Aug 15;8(8):e71770. doi: 10.1371/journal.pone.0071770 (PMC3744467; doi:10.1371/journal.pone.0071770)

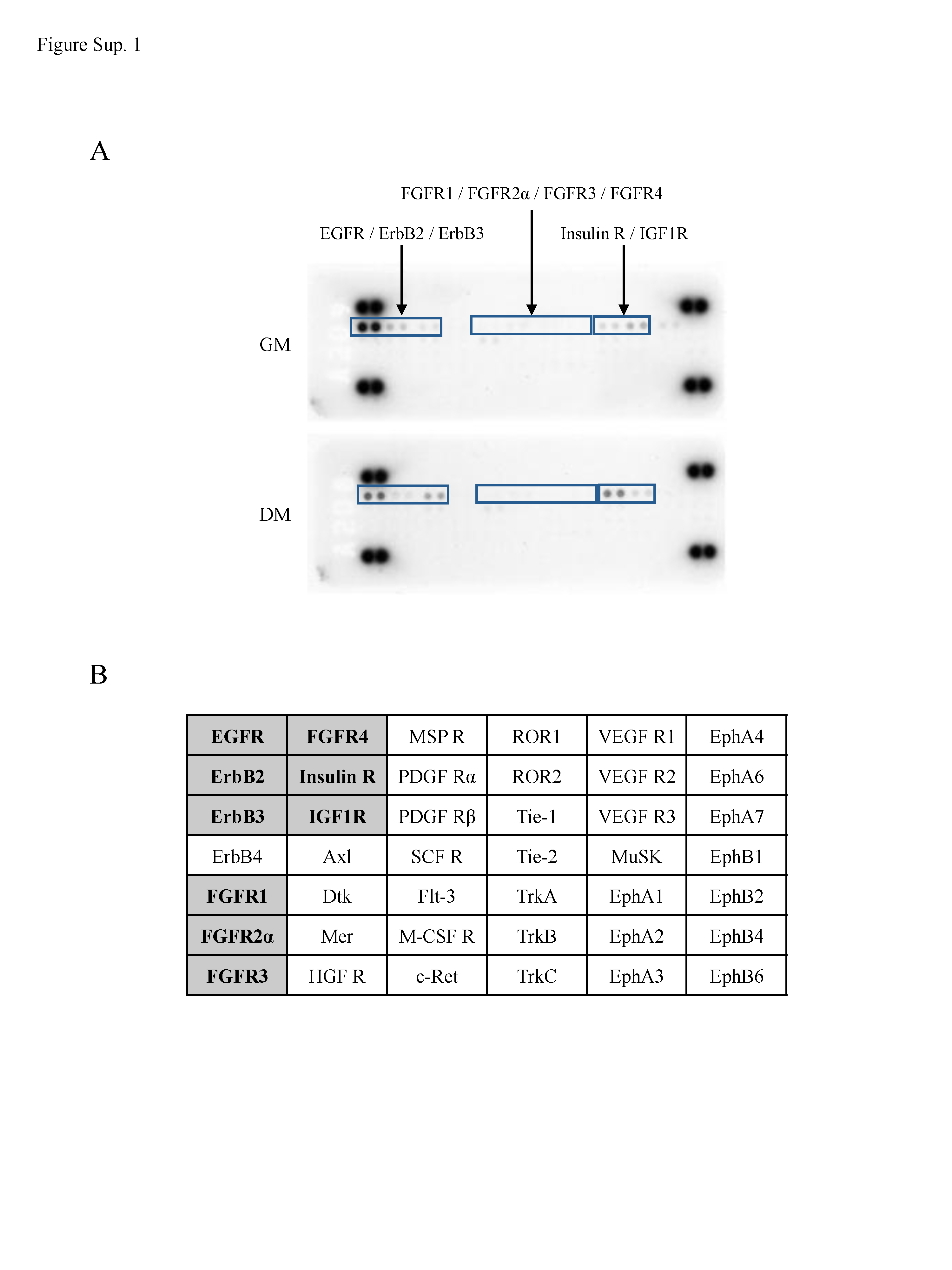

Supplement: Figure S1 — Receptor Tyrosine Kinases (RTKs) were tested for their activities in proliferation (GM) and differentiation conditions (DM 24 h). Panel A: Immunoblots of the RTK profiler array. Each pair of spots corresponds to a phosphorylated RTK. Panel B: List of the RTKs tested. (TIF) [file pone.0071770.s001.tif]
